# Revealing Plasma-Surface Interaction at Atmospheric Pressure: Imaging of Electric Field and Temperature inside the Targeted Material

**DOI:** 10.1038/s41598-020-59345-0

**Published:** 2020-02-17

**Authors:** Elmar Slikboer, Kishor Acharya, Ana Sobota, Enric Garcia-Caurel, Olivier Guaitella

**Affiliations:** 1 LPP, CNRS, Ecole Polytechnique, UPMC, IP, Paris, 91128 Palaiseau France; 2 Department of Applied Physics, EPG, Eindhoven University of Technology, Eindhoven, The Netherlands; 3 LPICM, CNRS, Ecole Polytechnique, IP, Paris, 91128 Palaiseau France

**Keywords:** Imaging and sensing, Applied physics, Plasma physics, Imaging techniques

## Abstract

The plasma-surface interaction is studied for a low temperature helium plasma jet generated at atmospheric pressure using Mueller polarimetry on an electro-optic target. The influence of the AC kHz operating frequency is examined by simultaneously obtaining images of the induced electric field and temperature of the target. The technique offers high sensitivity in the determination of the temperature variation on the level of single degrees. Simultaneously, the evolution of the electric field in the target caused by plasma-driven charge accumulation can be measured with the threshold of the order of 10^5^ V/m. Even though a specific electro-optic crystal is used to obtain the results, they are generally applicable to dielectric targets under exposure of a plasma jet when they are of 0.5 mm thickness, have a dielectric constant greater than 4 and are at floating potential. Other techniques to examine the induced electric field in a target do not exist to the best of our knowledge, making this technique unique and necessary. The influence of the AC kHz operating frequency is important because many plasma jet designs used throughout the world operate at different frequency which changes the time between the ionization waves and hence the leftover species densities and stability of the plasma. Results for our jet show a linear operating regime between 20 and 50 kHz where the ionization waves are stable and the temperature increases linearly by 25 K. The charge deposition and induced electric fields do not increase significantly but the surface area does increase due to an extended surface propagation. Additionally, temperature mapping using a 100 *μ*m GaAs probe of the plasma plume area has revealed a mild heat exchange causing a heating of several degrees of the helium core while the surrounding air slightly cools. This peculiarity is also observed without plasma in the gas plume.

## Introduction

Ever since cold atmospheric pressure plasmas were first applied for biomedical purposes^1^ there has been a tremendous amount of investigation to the interactions of these non-thermal discharges with various bio-surfaces^2–5^. In addition it initiated a regained interest in plasma liquid interactions^6^ and brought forth the field of plasma agriculture^7^. Furthermore, these types of discharges have also been used for surface functionalization of temperature sensitive materials^8,9^, flow actuators^10,11^ and plasma catalysis^12^. Cold atmospheric pressure plasmas are considered advantageous for these applications due to a combined presence of electric fields, (UV) radiation, moderate heat transfer and reactive oxygen and nitrogen species (RONS)^13,14^. The plasma target interaction mechanism is evidently important for the resulting effects that have been observed for those various applications.

The electric field caused by charge deposition on the targeted surface is an important parameter to monitor, since it influences the dynamics of the discharge propagation at the surface^15^. The electric field in the gas phase cannot simply be extrapolated due to surface charges and the material’s dielectric constant. The use of electro-optic materials as a target is, as far as we know, the only direct way to obtain the induced electric field. This has been applied already in the past for our plasma jet to investigate the influence of applied voltage and gas flow^16^ but the influence of operating AC frequency wasn’t looked at. In our previous works, a constant background birefringent pattern was subtracted to obtain the electric field results. We have later shown that this background pattern relates to the inhomogeneous temperature elevation of the sample^17^, which we use in this work to our advantage to simultaneous detect electric field and temperature dependence on the AC operating frequency.

The measurements of electric field and temperature inside targets directly exposed to a plasma are necessary with a high spatial and time resolution for electric field and very high accuracy about 1 degree for surface temperature. These challenging requirements can be achieved inside a dielectric by Mueller polarimetry.

Mueller polarimetry is used in this work to study to which level of intensity and spatial distribution of electric fields the studied samples are exposed to by a non-thermal plasma jet, by measuring the Mueller matrix of an electro-optic target. Electric fields measured with electro-optic crystals are due to surface charges deposited during the interaction, following the Pockels effect^18–21^. Recently it was found that simultanously with the electric field also information is obtained involving the temperature induced by the plasma by following the photo-elastic effect^17^. BSO (Bi_12_SiO_20_) is the electro-optic crystal used in this work with a dielectric constant of 56. This means that the studied plasma target interaction is characterized by this relatively high dielectric constant. Recent numerical work^22^ has shown that the experienced electric field inside targets of 0.5 mm thickness at floating potential are similar when the dielectric constant is higher than 4. This means that the reported electric field values and patterns in this work could be comparable to the electric field which is experienced inside e.g. a thin liquid layer, biological tissues, and polymers that are 0.5 mm when they are treated by a helium plasma jet and they are not grounded. Although, it is noted that cell models and biological tissues are more complex and hetrogeneous with a relative permittivity varying over a large range^23,24^. During the course of the measurements done in this work the BSO material has not shown signs of damage under the plasma exposure.

In the method section the calibration procedure is explained to show how the temperature spatial profile is obtained together with the induced electric field profile. This offers a unique diagnostic to investigate the plasma surface interaction. It has a significant advantage over conventional diagnostics where normally quantities are obtained inside the plasma in the gas phase and as such have to be extrapolated to estimate what the targeted materials experience at that instance. In our previous works, a comparison is made with a two dimensional fluid model^25,26^. This validates our technique and electric field results and shows that indeed the electric field in the gas phase and inside a dielectric target are different and should be measured separately. The measured temperature profile is calibrated and validated by comparing the temperature increase measured with a 100 *μ*m optical GaAs probe, as shown in the method section. The optical temperature probe is also used in this work to make a two dimensional map of the temperature profile in the plume area of the jet, showing how the mixing of the gas in to the surroundings causes a mild heat exchange, which is often overlooked.

This work will investigate the surface interaction induced by a non-thermal kHz-driven plasma jet. The influence of the operating AC frequency of the jet is examined in the kHz range. This parameter is important because it determines the time between consecutive ionization events. An introductory explanation of our plasma jet is given in the method section. When the operating AC frequency changes, the stability of the ionization waves can alter due to a change in left-over species densities. The effect this has on the targeted surface in terms of plasma surface propagation, charge deposition (an thus induce electric field) and temperature of the sample are reported in this work. Multiple jet designs are used throughout the world, all operating usually on different frequencies. The effect this has on the surface interaction is unknown and makes this work important.

## Results

### Temperature and electric field under plasma exposure

Mueller polarimetry is applied to examine the plasma surface interactions in terms of the electric field which is induced inside a target and the temperature pattern that is formed. It is used to study the effect of operating frequency of the AC plasma jet. The driving frequency is an important operating parameter since it determines the time in between the ionization events and as such influences the plasma dynamics.

**Figure 1:**
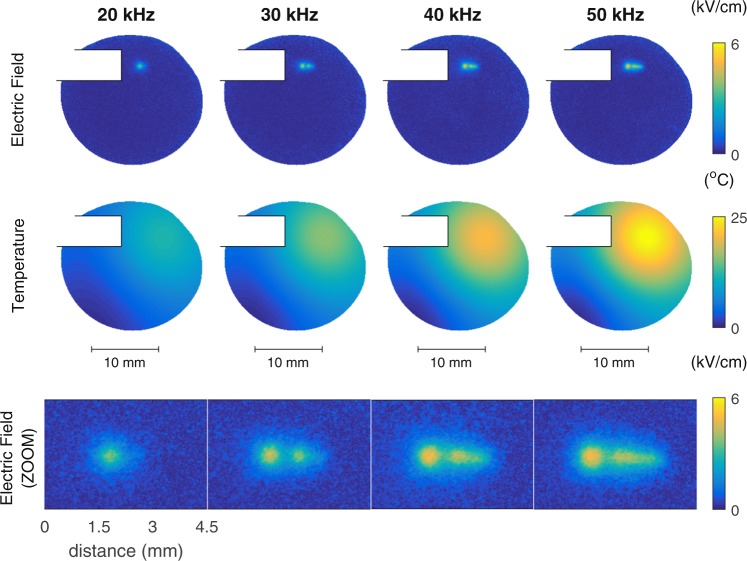
Images of the electric field patterns(top row) in kV/cm and temperature in °C obtained for different operating frequencies of the AC driven plasma jet^27^, using 1 slm helium and 2 kV voltage amplitude. An additional zoom for the electric field around the area of impact of the ionization waves is added below. The temperatures are shown relative to the room temperature. The capillary of the jet partially blocks the field of view.

The obtained electric field and temperature inside the targeted material as a function of operating frequency of the plasma jet are shown in Fig. 1. These results are part of the PhD thesis of Elmar Slikboer^27^. When the driving frequency of the AC applied voltage is increased, the maximum temperature increases (relative to the room temperature) and the shape of the electric field pattern changes as well as its maximum amplitude.

With an operating frequency of 20 kHz the amount of total heat generated by the plasma jet causes the target to have an increased maximum temperature of 11. 7 °C. With a higher operating frequency the amount of ionization events per time interval increases and as such, the generated heat causes the temperature in the target to increase to 24 °C at 50 kHz. The shape of the temperature profile reveals a non-symmetrical structure. This is because the heat produced by the plasma jet is carried by the helium flow, which spreads in a non-uniform way, since the plasma jet is impacting the surface at a 45 degree angle in addition to buoyancy forces acting on the lighter helium. The maximum temperature increase is observed in the impact point at the targeted surface of the ionization waves generated by the jet.

In this region the charges are deposited during the plasma-surface interaction and the electric fields are generated as a consequence. The electric field patterns that are formed are much smaller compared to the temperature profiles, and a zoom image is also shown on Fig. 1. When the operating frequency of the jet is increased, the amount of charge deposited on the surface increases and the resulting electric field pattern changes. A secondary surface discharge is observed after the impact point, in the direction of the helium flow (towards the right) on the surface. Similar behaviour is observed for a change of voltage or gas flow, as is shown in^16^. The material experiences electric fields due to surface charges of maximally 5.1 ± 0.2 kV/cm. This is an average throughout the 0.5 mm thick sample.

The electric field values shown here corresponds to the axial component of the field, perpendicular to the targeted surface. This perpendicular field is induced by charges deposited on the electro-optic BSO target and is much lower than the field measured in the plume of the same jet (typically 5 kV/cm in the target instead of 15–20 kV/cm in the plume^28^). This illustrates the need for direct measurement of field induced inside a target that cannot easily be extrapolated from the field values measured in the plasma. If desired, also the radial field components can be measured, either by examining the BSO crystal with the polarized light beam at an oblique angle or by using a different electro-optic crystal like Fe:LiNbO_3_^25,29^.

**Figure 2:**
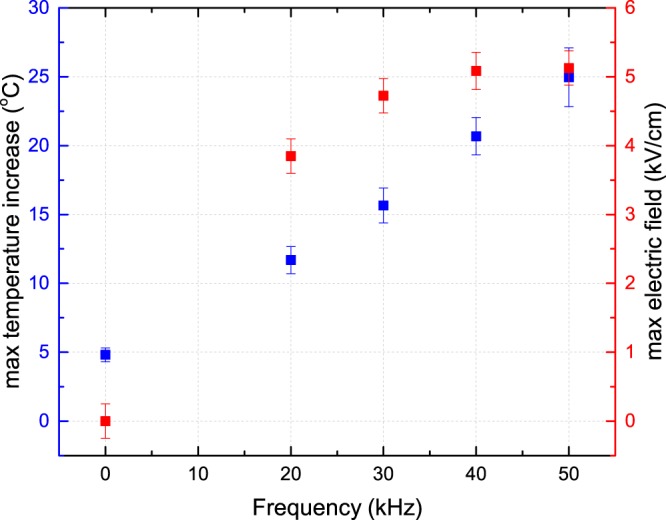
Maximum temperature increase (blue) on BSO relative to room temperature for different operating frequencies of the plasma jet together with the maximum (axial) electric field values (red).

Both the maximum electric field value and temperature increase observed in the obtained patterns are shown in Fig. 2. The uncertainty for the temperature (1–2 °C) relates to the standard deviation between the numerical temperature profiles obtained after repeating the fitting procedure to build the temperature profile from the measured birefringent patterns. The uncertainty for the electric field values (approximately 0.2 kV/cm) relates to the noise level in areas where no charge deposition has occurred and hence the electric field should be zero.

A small temperature increase is observed when no plasma is present (frequency of 0 kHz) while the helium flow is maintained at 1 slm. This is also shown in the method section when the voltage amplitude is varied at 30 kHz. Understandably, since no plasma is present, the measured electric field is zero. The examined electric fields are caused by surface charges deposited during the plasma surface interaction and the temperature increase is a result from transferred heat by the plasma jet. To investigate the heat delivered by the plasma further, the optical GaAs probe is used to map the temperature profile in the plasma plume, i.e. the region in between the capillary of the jet and the targeted surface. Results obtained using polarimetry and the GaAs probe agree well to each other.

### Temperature investigation in the plasma plume

**Figure 3:**
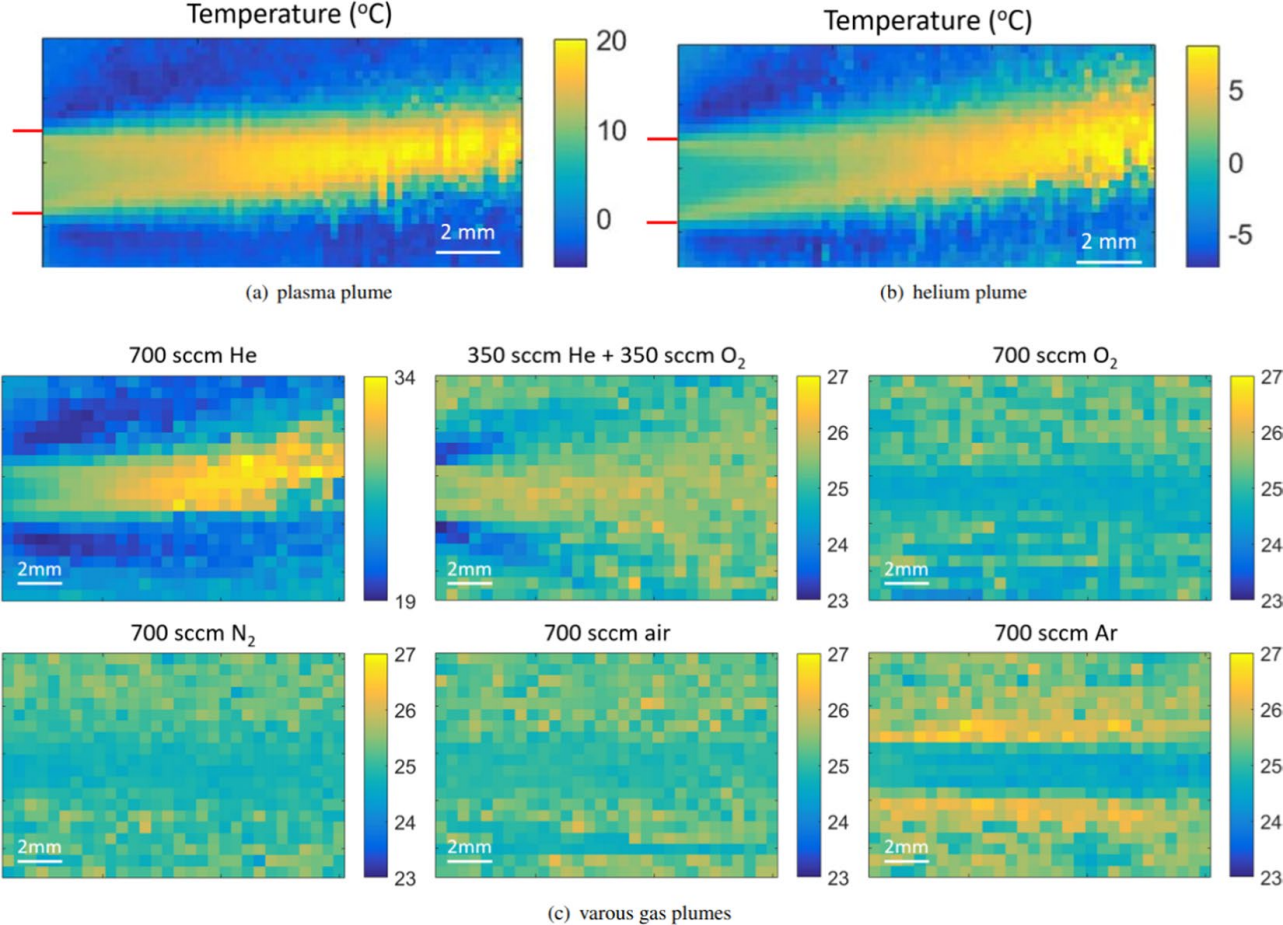
(**a**) Temperature variation profile of the plasma plume operated using a 1 slm helium flow and 2.0 kV voltage amplitude at 30 kHz AC. (**b**) Temperature variation profile of the gas plume using a 1 slm helium flow and 0 kV voltage amplitude (only gas flow). The exit of the capillary is indicated on the left border, resulting in a flow towards the right. The maximum increase is 18 °C relative to room temperature when the plasma is applied and 8 °C when only a helium gas flow is present. (**c**) Absolute temperature profiles (°C) in the gas plume (15 by 10 mm grid) using a 700 sccm flow and 0 kV voltage amplitude (only gas flow) for various carrier gasses, with a room condition of 25 °C. The exit of the capillary is again located at the left border. These results are a part of the PhD thesis of Slikboer^27^.

The heating caused by the plasma jet is examined further by investigating the temperature profile in the plasma plume by using the temperature probe sensor without the presence of a target. The profile of the temperature variation inside the target with respect to unperturbed air, mapped in a vertical plane through the axis of the plasma jet at the exit of the capillary, is shown in Fig. 3(a). The plasma jet is operated similarly as before at 30 kHz with a 2.0 kV amplitude using a 1 slm helium flow. In the figure, positive values indicate heating, negative values cooling, and null corresponds to no variation with respect to the unperturbed air.

The maximum temperature increase in the plume is 18 °C, while the surroundings have a lower temperature than ambient air, i.e. there is a 4 °C cooling effect. At approximately 7 mm from the exit of the capillary, the temperature in the freely expanding plume is similar to the temperature measured in the target at that distance. The visible length of the plasma plume at these conditions is maximally 1.0 cm. A temperature increase of 10 degrees Celsius is observed right at the exit of the capillary.

The cooling effect of the surrounding is more evident when the plasma is turned off and the temperature profile is measured with a 1000 sccm helium gas flowing out of the jet in to open air, see Fig. 3(b). Then the temperature increase at the exit of the capillary is zero, while the maximum in the gas plume is 8 °C. The surroundings are cooled to a temperature 6 °C below room temperature. This heat exchange occurring due to the helium-in-air mixing is remarkable, since the helium gas flows at low velocities (approximately 2.4 m/s) and the gas pressures remain at ambient (atmospheric) conditions, making it a stable laminar flow in these conditions as visualized by Schlieren imaging^30^. The formation of a clear boundary layer is observed at the edges of the capillary tube (indicated in red).

To investigate the origins of this heat exchange between the helium carrier gas and the surrounding air, different carrier gasses are examined. Fig. 3(c) shows the absolute temperature profile in °C of the gas plume for a 700 sccm gas flow of different types, i.e. helium, oxygen, nitrogen, (dry) air and argon, while no voltage is applied. These gases have varying densities, as well as specific heat. As a result there is a difference in heat exchange between the carrier gas and the surrounding air. Gas flow velocities are still relatively small and ambient pressure is at room conditions. For all cases the temperature at the exit of the capillary (left side) is equal to room conditions, i.e.  ~25 °C.

When 700 sccm helium gas flow is used the surroundings cool while the core where helium flows heats up. This effect is reversed when the heavier argon is used, though the effect is less significant. For air, nitrogen and oxygen as carrier gas changes in the temperature profile are insignificant, while still a flow of 700 sccm is applied.

## Discussion

In this work Mueller polarimetry is applied to investigate a plasma-surface interaction by measuring simultaneously the electric field and temperature in a target under exposure of the plasma jet when operating at different driving frequencies. Spatial imaging reveals that the plasma interaction with the surface covers a greater area in terms of temperature increase than induced electric field. The latter is local and depends on the location of deposited surface charge, while the observed in-homogeneous temperature distribution is due to heat generated by the plasma jet transported by the helium-in-air mixing.

This was done using a BSO crystal with a dielectric constant of 56 and a thickness 0.5 mm. We expect our electric field and temperature results to be valid for the interaction of non-thermal helium plasma jets with dielectric surfaces greater than 4 without a grounded plane in the vicinity. This has been shown numerically for the experienced electric field in a recent investigation^22^. The temperature of the target will vary with the volume of the dielectric, especially its thickness, because this determines how good the dielectric acts as a heat sink. As such for dielectric of comparable thickness the temperature results should be comparable as well. This means that even though living tissue and other biological samples are clearly too complex to be reduced to a plain dielectric for all the aspects of the interaction, for the induced electric field and the temperature elevation, the presented results are still applicable when the samples are 0.5 mm thick and far way from grounded planes.

In this work the effect of AC kHz operating frequency has been examined. Both the maximum value of electric field and temperature increase as a function of operating frequency of the plasma jet. For the induced electric field the increase in value is relatively minor ranging from 3.8 ± 0.2 to 5.1 ± 0.2 kV/cm, while the shape changes more significantly due to the appearance of the secondary surface discharge behind the impact point. The maximum temperature increase is found to scale linearly as a function of operating frequency.

This can be explained by the linear increase of the number of ionization events occurring per time interval, while heat produced per ionization event remains approximately the same. For this specific plasma jet, the linear behavior corresponding to the regime with one single reproducible ionization front during each period of the voltage is observed from 20 to 50 kHz. With higher frequencies the ionization waves are produced too quickly after each other, making them unstable. For lower frequencies there is too much time in between the ionization waves, which also induces an a-periodical behavior. Within this AC kHz regime, our results have been reproducible over many separate experiments since the ionization waves are reproducible in time and space.

Although the maximum electric field value does not change linearly within this identified regime, the surface pattern does seem to evolve in a linear way. The surface dynamics change with higher frequencies creating a spatially elongated surface interaction. The operating frequency changes the time in between the ionization events. Since spatial changes of the surface dynamics are observed, this stresses the importance and influence of leftover charges and species created in the previous power cycle. Less time in between the ionization events means that the left-over charge densities are higher, which assists the ionization wave in its propagation. This could explain the elongation of the surface patterns that are observed for higher operating frequencies.

The identification of the linear operating regime of a plasma device is important for several reasons. First of all, it allows comparison between several measurements that are not done at exactly the same conditions. Also, it is important for possible model-based feedback control of a plasma jet^31^.

To the best of our knowledge the only way to examine which electric fields are experienced by a targeted material under plasma exposure is by using electro-optic crystals and exploiting the Pockels effect. The electric field can also be examined in the gas phase, e.g. by using Stark spectroscopy^28,32^ or four-wave-mixing^33,34^, but this will only give you the electric field generated inside the plasma. Locally, this electric field will be relatively high (15-20 kV/cm) due to charge separation allowing for the propagation of the ionization wave, but this does not mean that a targeted material experiences the same quantitative fields, as is shown here with experienced electric fields of 5 kV/cm. The separated fields caused by either volume or surface charges have been examined in^25,26^. Due to charge deposition and alteration of the propagation dynamics in the vicinity of the dielectric target, the electric field values in the plasma plume cannot easily be extrapolated to determine what targets are experiencing. This makes our experiments as done as in this work crucial.

As of now the electric field inside "real” samples cannot be determined yet since the electro-optic properties of certain crystals like BSO have to be exploited, making this the only way to be able to study the induced electric field. As stated, the results are generally valid for dielectric targets with a relative permittivity greater than 4 of 0.5 mm thickness that are at floating potential.

Spatial profiles of the temperature obtained with Mueller polarimetry and the optical probe agree in both shape of the induced heating pattern and the induced temperature variation. Other approaches of examining the temperature induced by plasma focus again on the gas phase and will not give these accurate results. In general, gas or heavy particle temperatures can be estimated following different approaches, e.g. optical emission spectroscopy using rotational lines^35^, Raman or Rayleigh scattering^36^, Doppler broadening of metastables and optical (dielectric or metallic) probes. A comparison of several techniques done by *Hofmann et al*. shows the complications and inconsistencies that can arise^37^ in addition to the significant error bars that are unavoidable with some of the above-mentioned diagnostics.

The maximum induced temperature difference relative to ambient conditions obtained from the spatially obtained profiles reported in this work are comparable with other non-thermal jets where only the maximum temperature is reported. *Weltmann et al*. measured the temperature in the plasma plume of their RF-driven plasma jet (kINPen 09, INP Greifswald, Germany)^38^ and found a temperature upto 63 °C. In a differently designed RF-driven plasma jet gas temperatures are observed of several hundreds of degrees^39^, while temperature measurements in the plasma plume of an AC kHz-driven plasma jet show only a gas temperature of 35 °C^28^. Numerical and experimental work done by *Kelly et al*. on the micro scaled reference jet show a great variance of gas temperature depending on the working mode of that jet^40^. The temperature increase due to the heat exchange between the heated helium core and the cooled surrounding air has not been observed and reported in literature before to the best of our knowledge.

The temperature induced by the plasma relates to the theoretical value of 23 °C, calculated using the dissipated power of the plasma jet (operated at 30 kHz and 2.0 kV) of 0.2 W^41^ used solely to heat a volume of helium defined by the 1000 sccm gas flow. The mapping of the plasma plume using the temperature probe reveals that only an increase of 10 °C is reached inside the capillary tube of the plasma jet. This is most likely related to Ohmic heating due to elastic collisions in the region in between the electrodes. The temperature profile increases further downstream outside the jet due to the plasma plume and the newly found heat exchange caused by the helium-in-air mixing. The resulting temperature profile where the maximum is located downstream instead of at the exit of the capillary is often not incorporated in numerical models. This is important to take properly into account the effect of local heating in the modification of gas flow dynamic induced by plasma jet as discussed in^42,43^. The observed heat exchange was also present without any plasma (0 kV or 0 kHz) while still operating a constant 1 slm helium flow.

The examined gas plumes do not have an increased temperature at the exit of the capillaries. However, an increase is observed downstream along the core of the helium gas flow. This is accompanied with a cooling of the surrounding air. This heat exchange outside the capillary is not well understood since gas velocities are low and ambient pressure conditions are maintained, but it is related to a difference in either or both density and specific heat of the core carrier gas compared to the environment. With lighter helium, the core heats up while the environment cools. On the contrary, with heavier argon as carrier gas this effect is opposite. When nitrogen, oxygen or air is used as carrier gas of the jet (without an applied voltage) there is no heat exchange.

The additional heat exchange between the helium core and the surrounding air complicates the discussion of flow alterations due to the plasma jet. From Fig. 3(a,b) it can be seen that the helium flow tends to rise up as a consequence of buoyancy force. However when the plasma is on, the rising of the flow at a given distance is less than without plasma (about 1 mm instead of 1.8 mm without plasma at 16 mm distance from the nozzle). The modification of the gas flow dynamics by the plasma is a well known fact and several observations have been made stressing the importance of heating and/or ion wind, causing these changes in the helium flow^42,44–48^. No consensus has been reached yet whether heating or ion-wind is dominant. The contribution of the increase in temperature to the gas flow modification is usually estimated from the change in helium density with temperature and the continuity of mass flow rate just at the nozzle^44^. However Fig. 3(a) shows that the maximum temperature increase occurs at more than 10 mm after the nozzle. This is important to take into account for a proper estimate of the gas velocity increase due to lowering of gas density with temperature. Unfortunately, the simple approach of using the continuity equation of the mass flow rate is then difficult to apply since the cross section of the gas stream becomes poorly defined.

The newly applied optical diagnostic technique has been applied to reveal plasma target interactions occurring in terms of the electric fields and temperature profiles induced *inside* a targeted electro-optic material under exposure of a plasma jet. This examination allowed for the simultaneous imaging of the electric field and temperature generated inside a material, instead of measuring it in gas phase and extrapolating it towards the target. This has never been done before in this way and opens new possibilities to investigate spatially and time-resolved the plasma surface interactions that occur.

## Methods

### Atmospheric pressure plasma jet

**Figure 4:**
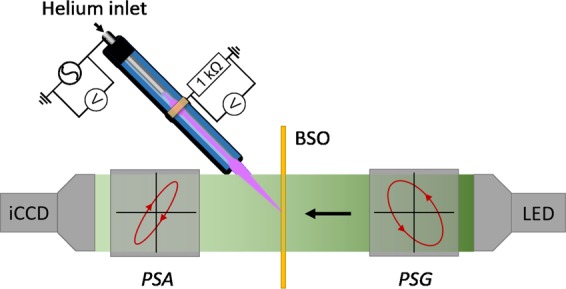
The experimental setup used to measure the Mueller matrix of the BSO crystal under exposure of the plasma jet (enlarged view of the jet compared to the other elements), showing the Mueller polarimeter consisting of a light source, *PSG* (Polarizer State Generator), *PSA* (Polarizer State Analyzer) and the iCCD detector.

The plasma target interaction investigated in this work is done for discharges generated with a plasma jet operated in a coaxial configuration examined with a Mueller polarimeter, see Fig. 4. Helium flows at 1 slm through an inner stainless steel tube, acting as powered electrode (30 kHz AC with 2.0 voltage amplitude). This is surrounded by an outer dielectric pyrex tube. A grounded copper ring is attached around the pyrex capillary at 5 mm from the end of the powered electrode. Towards the end of the capillary (inner diameter of 2.5 mm and outer diameter of 4 mm) the distance is 20 mm. A more comprehensive description of this plasma jet can be found in^27,41^. The amount of power dissipated by the jet is approximately 0.2 W. The jet impacts the surface at a 45° angle at 7 mm distance^41^.

When the plasma jet is operated using a 30 kHz driven AC signal, there is one ionization wave generated during every positive half period travelling away from the powered electrode^41^. In proximity of a target, charge will be deposited on impact of the ionization wave. This is removed approximately 10 *μ*s later when the polarity of applied voltage changes, observed through electric field imaging^15^. This is accompanied by a weak afterglow, indicating the occurrence of a (back) discharge at the target going back towards the capillary tube.

### Time-resolved mueller polarimeter

The temperature and electric field induced in a target by the plasma jet is investigated using Mueller polarimetry. In this novel optical technique polarized light is sent through an electro-optic BSO crystal for which the refractive index depends on both the electric field and the temperature gradients^17^. Due to the examination of the material the plasma jet is positioned at a 45 degree impact angle, in order not to block the polarized light that is send through the target, see Fig. 4. The experimental setup is shown with more detail in^17,27^.

The Mueller matrix of the targeted electro-optic BSO crystal is measured by generating and analyzing different states of polarized light, using the Polarizer State Generator (*PSG*) and the Polarizer State Analyzer (*PSA*). Both the PSG and PSA consist of a polarizer and two ferro-electric liquid crystals, for which the orientation can be switched between two states. Hence, 16 different states can be sequentially examined which allows for the acquisition of the 4 × 4 Mueller matrix of the examined sample, i.e. the electro-optic BSO under exposure of the plasma jet.

By measuring the full Mueller matrix of the sample the birefringence is examined, following the logarithmic decomposition^49,50^. Diattenuation and depolarization are examined automatically as well, which opens interesting possibilities for future *in-situ* examinations of temperature sensitive targets directly. The acquisition of the polarized light can be timed within the applied AC period for time resolved Mueller polarimetry. The optical characterization of this highly dynamical system is not possible with a conventional static Mueller polarimetry setup. Since time resolved measurements are necessary for this work, an automatic control of the PSG and PSA states synchornized with the iCCD camera has been implemented.

The Mueller matrix is obtained twice while the plasma jet is active. Once before a new ionization wave has impacted and once right after, with an exposure time of 1 *μ*s. This is achieved by sychronizing the switching of the polarization states and the acquisitions at the iCCD camera with an external trigger, related to the rise of the positive half period of the applied high voltage AC sine wave. A more comprehensive description of the acquisition and theory behind the time resolved imaging Mueller polarimeter can be found in^17,27^ but we remind some of the key points here.

### Analysis of birefringent properties

When light travels through a birefringent material the wavecomponents will experience a relative retardance due to a difference in refractive index. The linear retardance which is induced depend on the polarization direction of the light, hence a difference can be induced for light which is linearly polarized either horizontally/vertically or diagonally, following the Stokes formalism of polarization.

This linear retardance Γ scales with the electric field and temperature gradient according to equation 2 as derived in^17^, with *d* = 0.5 mm the thickness of the BSO crystal, *n*_*o*_ = 2.54 the unperturbed refractive index and *λ* = 530 nm the wavelength of light. Two different Γs are obtained using Mueller polarimetry. The first, Γ_1_, corresponds to linear retardance induced between the vertical and horizontal polarized light components (defined by the *x* and *y*-axis), while the second Γ_2_ relates to the polarization components in the diagonal axes (i.e. the *x* − *y* and *x* + *y*-axis). They depend on the difference of experienced refractive indices in those respective coordinate systems, namely Δ*n*_0∕90_ and Δ*n*_45∕135_.

Γ_2_ scales with the electric field *E* parallel to the optical axis *z* according to the electro-optic coefficient *r*_41_ = 4.8 pm/V. The dependencies of Γ with the second derivatives of the temperature have to be determined through calibration, i.e. *γ*_1,2,3_. This is because they depend on the photo-elastic tensor elements of BSO, which are not so-well determined unlike the electro-optic coefficient. Γ is space dependent on *x* and *y* since both the electric field and temperature are non-homogeneous patterns.

1 $${\Gamma }_{1}={\Gamma }_{{1}_{E}}+{\Gamma }_{{1}_{T}}\ \ \ \ \,{\rm{and}}\,\ \ \ \ {\Gamma }_{2}={\Gamma }_{{2}_{E}}+{\Gamma }_{{2}_{T}}$$

2 $$\left\{\begin{array}{l}{\Gamma }_{{1}_{E}}=\frac{2\pi d}{\lambda }\Delta {n}_{0/9{0}^{{\rm{o}}}}=0\\ {\Gamma }_{{2}_{E}}=\frac{2\pi d}{\lambda }\Delta {n}_{45/13{5}^{o}}=\frac{2\pi d}{\lambda }{n}_{o}^{3}{r}_{41}{E}_{z}\end{array}\right.\ \ \ \ \,{\rm{and}}\,\ \ \ \ \left\{\begin{array}{l}{\Gamma }_{{1}_{T}}=\frac{2\pi d}{\lambda }{n}_{o}^{3}\left({\gamma }_{1}\frac{{\partial }^{2}T}{\partial {x}^{2}}+{\gamma }_{2}\frac{{\partial }^{2}T}{\partial {y}^{2}}\right)\\ {\Gamma }_{{2}_{T}}=\frac{2\pi d}{\lambda }{n}_{o}^{3}{\gamma }_{3}\frac{{\partial }^{2}T}{\partial x\partial y}\end{array}\right.$$

#### Electric field analysis

Twice the Mueller matrix is obtained time-resolved while the plasma jet is continuously operated, one timed right before the ionization wave impacts the target and once right after. By subtracting the birefringences obtained before and after impact the electric field effect is separated from the temperature effect. This is because the electric field is only present after impact due to charge deposition, while a steady state temperature profile is present at both times. The electric field is then easily obtained by applying the inverted equation 2.

#### Temperature analysis

For the retrieval/analysis of the temperature pattern it is less trivial since the birefringence depends on the second spatial derivative of the temperature profile, as shown in equation 2. A procedure is followed to calculate a temperature profile for which the second derivatives match the birefringence that is measured. A priori an asymetrical Gaussian-like temperature profile is expected due to the local heat delivered by the plasma jet in the impact region and the helium-in-air mixing. The main idea of the procedure is to build a temperature profile iteratively. Each step the temperature is slightly varied for a randomly proposed area which is only accepted if it improves the matching between the second derivatives of the numerically created profile with the measured birefringence. This is repeated several times to create an average profile and a characteristic for the uncertainty based upon the standard deviations between the repeated outcomes. The procedure is more comprehensively discussed in^17,27^.

### Secondary GaAs optical temperature probe

A second method for the examination of the temperature is used to verify the procedure to retrieve the temperature profile from the measured birefringent patterns. This is done using a 100 *μ*m GaAs temperature probe which is attached to an optical fiber (OpSens, model OTG-F). Since no metallic parts are present and the size of the probe is significantly smaller than the plasma jet, perturbations are expected to be low. This is monitored through voltage–current waveforms. The temperature profile can be mapped pointwise by changing the position of the probe along a predefined grid, by capturing the local temperature at each point. It has been shown in^17^ that the obtained inhomogeneous pattern using the birefringent patterns acquired with Mueller polarimetry strongly relates with the mapped temperature profile on the backside of a targeted material. The maximum induced temperature difference relative to the surroundings can be used to calibrate the values of *γ*_1,2,3_.

### Calibration fitting procedure

**Figure 5:**
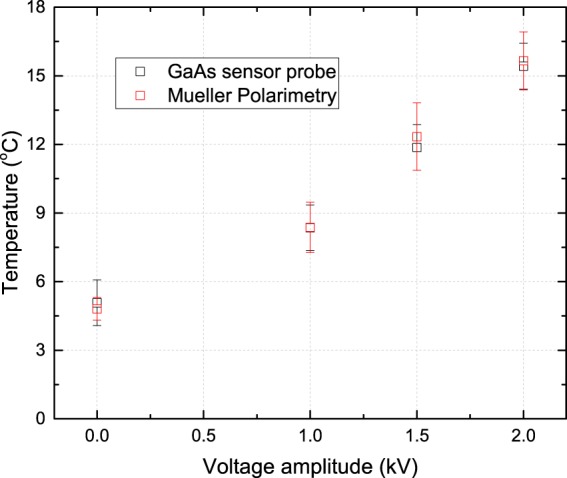
The maximum temperature increase on glass relative to room temperature for varied applied voltage amplitudes, shown in black. These data points obtained with the GaAs temperature probe sensor are used for the calibration of the Mueller polarimetry procedure to obtain the temperature profile from the birefringent patterns, shown in red.

From the numerical fitting procedure, two dimensional images of the temperature are obtained. The shape of the temperature profile is dependent upon the matching of the second gradients with the measured profiles. The maximum value of temperature increase can be controlled using the values of *γ*_1,2,3_. This procedure is calibrated by fixing *γ*_1,2,3_ by comparing temperature measurements obtained with Mueller polarimetry with measurements done with the temperature probe. By comparing the maximum temperature increase for different applied voltages of the plasma jet, the values of *γ*_1,2,3_ are optimized and calibrated, as shown in Fig. 5.

The best match for the maximum temperature increase when applying different voltage amplitudes between the numerical temperature profile obtained from Mueller polarimetry and the mapping using the probe is obtained for *γ*_1_ = −4 ⋅ 10^−12^, *γ*_2_ = 5.4 ⋅ 10^−12^ and *γ*_3_ = 10.8 ⋅ 10^−12^ m^2^∕K. A positive or negative sign is needed to match the polarity of the birefringent patterns to the second gradients of a temperature profile. The variation of the values of *γ*_1,2,3_ can be found in^27^.

The mapping with the temperature probe is done on the backside of a 0.5 mm thick glass under exposure of a plasma jet operated in similar conditions as when examining BSO with the Mueller polarimeter. It has been confirmed with the temperature probe on the backside in the impact region that similar values are obtained with a glass target as with BSO. This is understandable since the temperature increase is caused by heat delivered from the plasma jet.

Besides a good agreement between the two methods (with the calibrated values of *γ*_1,2,3_) Fig. 5 also shows that the maximum temperature increase scales linearly with the voltage amplitude. This is explained by an increase of input power. When operating the plasma jet at "normal” conditions, i.e. a voltage amplitude of 2.0 kV the temperature increases inside the target by 15.5 ± 1. 0 °C. When the voltage amplitude is set to 0 kV, i.e. with no plasma present but still a helium flow of 1 slm, there remains a minor increase in temperature of 5.0 ± 0. 9 °C.
